# Antioxidant Effects of *Sophora davidi* (Franch.) Skeels on d–Galactose–Induced Aging Model in Mice *via* Activating the SIRT1/p53 Pathway

**DOI:** 10.3389/fphar.2021.754554

**Published:** 2021-12-06

**Authors:** Beibei Lin, Dingqiao Xu, Sanqiao Wu, Shanshan Qi, Youmei Xu, Xiang Liu, Xiaoying Zhang, Chen Chen

**Affiliations:** ^1^ Chinese-German Joint Laboratory for Natural Product Research, College of Biological Science and Engineering, Shaanxi University of Technology, Hanzhong, China; ^2^ Key Laboratory of Shaanxi Administration of Traditional Chinese Medicine for TCM Compatibility, Shaanxi University of Chinese Medicine, Xi’an, China; ^3^ Centre of Molecular and Environmental Biology, Department of Biology, University of Minho, Campus de Gualtar, Braga, Portugal

**Keywords:** SIRT1, p53, D-galactose, anti-aging, *Sophora davidi* (franch.) skeels fruits extract

## Abstract

This study investigated the protective effect of *Sophora davidi* (Franch.) Skeels fruits extract (SDE) on d–galactose–induced acute aging in mice. Ultra performance liquid chromatography coupled with tine-of-flight mass spectrometry (UPLC-Q-TOF/MS) was performed to identify the composition of compounds in SDE. KM mice were divided stochastically into the normal control group (NC, saline), d–galactose (D-gal) model group, vitamin C (Vc) group (positive control), low–, medium–and high–dose SDE treat groups. After 28 days administration and fasting overnight, the serum, liver, and brain samples of mice were collected. The levels of inducible nitric oxide synthase (iNOS), acetylcholinesterase (AChE) activity in the brain, malondialdehyde (MDA) and reduced glutathione (GSH) content, superoxide dismutase (SOD) and total antioxidant capacity (T–AOC) activity in the liver and brain were measured. Immunohistochemistry was applied to detect silent information regulator 1 (SIRT1) and p53 protein expression in the liver and brain, and quantitative real-time polymerase chain reaction (qRT-PCR) was used to detect the expression of nuclear factor κB (NF–κB), tumor necrosis factor (TNF–α), interleukin–6 (IL–6), interleukin-1β (IL–1β), and anti-aging factor Klotho in the liver and brain. The results showed that UPLC-Q-TOF/MS identified 78 compounds in SDE. SDE could reduce the iNOS activity in serum and AChE activity in the brain, upregulate the levels of SOD, T–AOC and GSH in liver and brain, and debase the MDA content in liver and brain. SDE could downregulate the mRNA expressions of TNF–α, NF–kB, IL–1β, and IL–6 in the liver and brain, and elevate the mRNA expression of Klotho. SDE improved the pathological changes of the liver and brain induced by D–gal, increased the expression of SIRT1 protein in the liver and brain, and inhibited the expression of p53 protein induced by D–gal. To summarize, SDE demonstrated clear anti–aging effect, and its mechanism may be relevant to the activation of the SIRT1/p53 signal pathway.

## Introduction

Aging is a progressive, physiological impairment involving various organs and tissues, which can lead to normal cell regulatory dysfunction; can affect nervous system, respiratory system, immune system and other systems; and is a risk factor for many chronic diseases, such as cancers, cardiovascular diseases, and neurodegenerative diseases (Zhu et al., 2017; Bektas et al., 2018; Mahmut et al., 2020). With the increase of aging population and life expectancy, screening on natural and synthetic bioactive constituents with potential anti-aging pharmacological activity acquires research priority.

The construction of aging mice by long–term administration of D–gal is a classic model in aging related study (Zhou et al., 2013; Li et al., 2015). d–galactose (D–gal) is a type of reduced aldose, which exists naturally in the body, including the brain (Nagy and Pohl, 2015; Qing et al., 2018; Zhao et al., 2020; Mu et al., 2021). Under normal conditions, D–gal is metabolized into glucose, however, the host will produce excessive reactive oxygen species (ROS) and increase the oxidative stress under the challenge of excessive amount of D–gal. This pathological change can be used for the development of aging animal model for pharmacological investigation, as ROS will destroy the dynamic balance of oxidation and antioxidation, reduce the activity of antioxidant enzymes in the body, damage mitochondria and neurons, and cause cognitive, learning, and memory disorders, and aging phenomenon (Zhou et al., 2013; Kong et al., 2018).

*Sophora davidi* (Franch.) Skeels (*S. davidi*) is a semi–evergreen deciduous shrub species of the Leguminosae family, it has been used in the treatment of sore throat, hematochezia, lung heat cough, dysentery, gonococcal disease, edema, hematuria, and so forth (Tai et al., 2011; Huang et al., 2018; Lin et al., 2019). *S. davidi* is widely distributed, with dense flowers and light aromatic taste. Therefore, *S. davidi* is one of the main honey source plants in China. The flower, leaves, stem, fruit and root of *S. davidi* contain polyphenols, flavonoids, alkaloids, and other active substances that these components have anti–inflammatory, antitumor, hypoglycemic, and antioxidant effects (Ping et al., 1999; Wang et al., 2016; Xie et al., 2017; Lin et al., 2019). Previous reports indicated that polyphenols, flavonoids, alkaloids from *S. davidi* have anti-aging effects (Rosa et al., 2009; Ma et al., 2018; Hano and Tungmunnithum, 2020). Our previous studies have proved that the *S. davidi* extract showed good anti–oxidation activity *in vitro* (Lin et al., 2019), this study aimed to explore the anti–aging effect of *S. davidi* fruits extract (SDE) against D–gal–induced acute aging mice.

## Materials and Methods

### Materials

The fruits of *Sophora davidi* (Franch.) Skeels were gathered in the south of Qinling Mountains in September 2017 (110°54′ east longitude, 33°32′ north latitude), and authenticated by Prof. Sanqiao Wu from the College of Biological Science and Engineering, Shaanxi University of Technology.

d–galactose, rapid extraction kit of total RNA and ascorbic acid (Vc) were purchased from Sangon Biotech Co., Ltd (Shanghai, China). AChE, iNOS, MDA, SOD, T–AOC and GSH kits were purchased from Jiancheng Bioengineering Institute (Nanjing, China). BSA protein assay kit was purchased from Beyotime Biotechnology (Shanghai, China). DAB reagent kit was purchased from Zhongshan Jinqiao Biotechnology Co., Ltd (Beijing, China). SIRT1 antibody and p53 antibody were acquired from Biosynthesis Biotechnology Co., Ltd. (Beijing, China). cDNA reverse transcription kit and PCR kit were purchased from Takara Biomedical Technology Co., Ltd. (Dalian, China). Hematoxylin, eosin, and immunohistochemistry pen were obtained from Dingguo Changsheng Biotechology Co., Ltd. (Beijing, China).

### Preparation of SDE

The fruits were dried at 45°C and then smashed, a total of 40 ml ethanol (60%) was added to the smashed fruits (1 g) (fruit: solvent = 1 : 40, g: mL) for ultrasonic wave extraction under 100 W power (KQ5200DE CNC ultrasonic instrument, Jiangsu, China) for 30 min, subjected to suction filtration, and the residue was extracted repeatedly once. The two extracts were combined and concentrated by rotary evaporator. The resulting mixtures were filtered and dried in a lyophilizer. The SDE extract ratio is 1:0.257 (g: g). The plant extracts were kept at 4°C until further analysis.

### UPLC-Q-TOF/MS Analysis

The sample analysis was performed with a Waters Acquity™ ultra-performance liquid chromatography (UPLC) system (Waters Corporation, Milford, MA, United States) coupled with a Synapt G2 mass spectrometer (MS; Waters Corp, Manchester, United Kingdom) equipped with an electrospray ion (ESI) source. An Acquity UPLC BEH C_18_ column (2.1 × 100 mm, 1.7 mm) was applied for all analyses. The mobile phase was composed of A (0.1% formic acid water solution) and B (acetonitrile) with a gradient elution: 0–2 min, 5% B; 2–3 min, 5%–15% B; 3–4 min, 15%–25% B; 4–5 min, 25%–26% B; 5–6 min, 26%–35% B; 6–7 min, 35%–60% B; 7–8.5 min, 60%–95% B; 8.5–13 min, 95% B; 13–14 min, 95%–15% B; 14–15 min, 15%–5% B. The flow rate was set at 0.3 ml/min. The column temperature was set at 35°C. The detector was PDA and detection wavelength was 200–400 nm. Mass spectrometry detection was performed using an electrospray ionization source (ESI), positive and negative ion mode detection. The conditions of MS analysis were designed with positive as follows: the capillary voltage at 3 kV, the desolvation gas flow rate set to 600 L/h at a temperature of 350°C, the cone gas flow rate set at 50 L/h and the source temperature at 100°C. The scan range was 50–1,200 (*m/z*). The conditions of MS analysis were designed with negative as follows: the capillary voltage at 2 kV, the desolvation gas flow rate set to 600 L/h at a temperature of 350°C, the cone gas flow rate set at 50 L/h and the source temperature at 100 °C. The scan range was 50–1,200 (*m/z*). The data was acquired through Waters MassLynx v4.2 software (Waters Corporation, Milford, MA, United States)

### UPLC Quantitative Analysis

The quantitative analysis of SDE was performed on a Thermo UltiMate 3000 UPLC system (Thermo Scientific, Waltham, MA, United States) with diode-array detector (DAD). For the content determination of polyphenols according to the method described our previously report (Lin et al., 2019). The Inertsil/WondaSil C_18_ phase-HPLC column (250 mm × 4.6 mm i. d, 4 μm particles) was used to separate and quantify individual alkaloids, and the detection wavelength is 205 nm. Acetonitrile (A), 0.05 M potassium phosphate monobasic (B, acetic acid adjusted to pH 4.5) and ultrapure water (C) were used as mobile phase. The following linear gradient elution: 0–30 min, 2:88:10%–3:87:10% (A: B: C) was used to analyze samples. The injection volume was 10 μL with 1 ml/min flow rate. The detection temperature is 35°C. Each sample was paralleled three times.

### Animals and Treatments

A total of 48 male and female Kunming mice (SPF level) were obtained from Chengdu Dasuo experimental animal Co., Ltd (Chengdu, China). The mice with weight of 22 ± 5 g, were kept at 25 ± 2°C with a humidity of 55 ± 5%, and 12 h light and dark cycle. They were allowed to access to feed and water freely and were fed adaptively during the experiments. Mice were divided into six groups (*n* = 8): normal control group (treated with normal saline everyday); D–gal model group (treated with D–gal 200 mg/kg BW/day); low–, medium–, and high–dose groups (SL, SM, and SH group, treated with SDE 125, 250, and 500 mg/kgBW/day, respectively); and Vc positive control group (treated with D–gal, Vc 50 mg/kg BW/day). In the next 4 weeks, the growth and mental state of mice were observed. After 28 days into the experiment, the mice were fasted overnight, weighed and then their blood, liver and brain were collected. Liver and brain were weighed and the organ indexes were calculated. The blood was centrifuged at 3,000 rpm at 4°C for 10 min to obtain the serum, which was then kept at –80°C for further analysis. Parts of the liver and brain of each mouse were fixed in 4% paraformaldehyde solution for histomorphology observation. The remaining liver and brain tissues were stored at –80°C.

All animal procedures were performed in accordance with the Animal Ethics Committee of Shaanxi University of Technology (2019–007, Chinese–German Joint Laboratory for Natural Product Research).

### Histomorphological Observation of Liver and Brain

The mice liver and brain tissues fixed with paraformaldehyde solution were embedded in paraffin, then sectioned with a 5 μm tissue slicer (Leica, Wetzlar, Germany) and stained with hematoxylin–eosin (H&E). The pathological changes of the tissues were observed under microscope (Leica, Wetzlar, Germany) (Li et al., 2019).

### Determination of iNOS Activity in Serum

The activity of iNOS in serum was measured by ELISA kit according to the manufacturer’s instructions.

### Measurement of the Biochemical Indicators in Liver and Brain of Mice

To determine the activity of AChE in the brain and the activity of SOD, T–AOC, the content of GSH and MDA in the liver and brain, we took the liver and brain tissue, added ice and normal saline in the proportion of 1:9, homogenate (OMNI, Kenneesaw, GA, United States), centrifuged at 4°C, 3,000 rpm for 10 min, and then took the supernatant and packed it separately at –20°C for storage (Zhao et al., 2018).

### Immunohistochemistry

The expressions of SIRT1 and p53 proteins in the liver and brain were detected by immunohistochemistry. The liver and brain sections were dewaxed with xylene and gradient alcohol, triton X–100 broke the membrane for 30 min, followed by incubation with 3% H_2_O_2_ for 30 min, 3% BSA sealed for 20 min, anti–SIRT1 and anti–p53 antibody incubated for 2 h (1:250), horseradish perioxidase (HRP) incubated for 1.5 h (1: 200), DAB stained (liver 20 min, brain 10 min), neutral gum sealed and counterstained with hematoxylin. The expressions of SIRT1 and p53 protein in liver and brain of mice in each group were analyzed and photographed under the same setting.

### Quantitative Real–Time PCR

Rapid extractions of total RNA from liver and brain tissues were conducted with a total RNA extraction kit. The first-strand cDNA was reverse-transcribed using the PrimeScript RT reagent kit. The transcription levels of TNF–α, NF–kB, IL–1β, IL–6, and Klotho were quantified by qRT-PCR with GAPDH Gene as internal reference. The qRT-PCR was analyzed on real time PCR detection system with SYBR green (StepOnePlus, ABI, Carlsbad, CA, United States). The primer sequences of the targets and reference genes are summarized in Table 1.

**TABLE 1:** qRT-PCR amplification primer.

Target gene	Upstream primer (5′–3′)	Downstream primer (5′–3′)
TNF–α	TAT​GGC​TCA​GGG​TCC​AAC​TC	GCT​CCA​GTG​AAT​TCG​GAA​AG
NF–kB	ACG​ATC​TGT​TTC​CCC​TCA​TCT	TGG​GTG​CGT​CTT​AGT​GGT​ATC
IL–1β	TCC​AGG​ATG​AGG​ACA​TGA​GCA​C	GAA​CGT​CAC​ACA​CCA​GCA​GGT​TA
IL–6	CAA​AGC​CAG​AGT​CCT​TCA​GAG	GTC​CTT​AGC​CAC​TCC​TTC​TG
Klotho	GGG​ACA​CTT​TCA​CCC​ATC​ACT	TGG​GTG​CGT​CTT​AGT​GGT​ATC
GAPDH	ACA​GTC​CAT​GCC​ATC​ACT​GCC	GCC​TGC​TTC​ACC​ACC​TTC​TTG

After qRT-PCR, the relative expression level of each gene was calculated using GAPDH as the internal reference gene (Li et al., 2019), and the formula was 2^–(△△Ct)^.

#### Statistical Analysis

Data were analyzed using SPSS 19.0 software (SPSS Inc, Chicago, IL, United States). GraphPad Prism 5 (GraphPad Software, San Diego, California, United States) was used for drawing. Mass spectral data were collected and analyzed using MassLynx V 4.2 software (Waters, Milford, MA, United States). The one–way analysis of variance (ANOVA) with Duncan’s multiple range tests and *p* < 0.05 was considered statistically significant. Three repetitions in each experiment were displayed as mean ± standard deviation (SD).

## Results

### Chemical Composition of SDE

**FIGURE 1:**
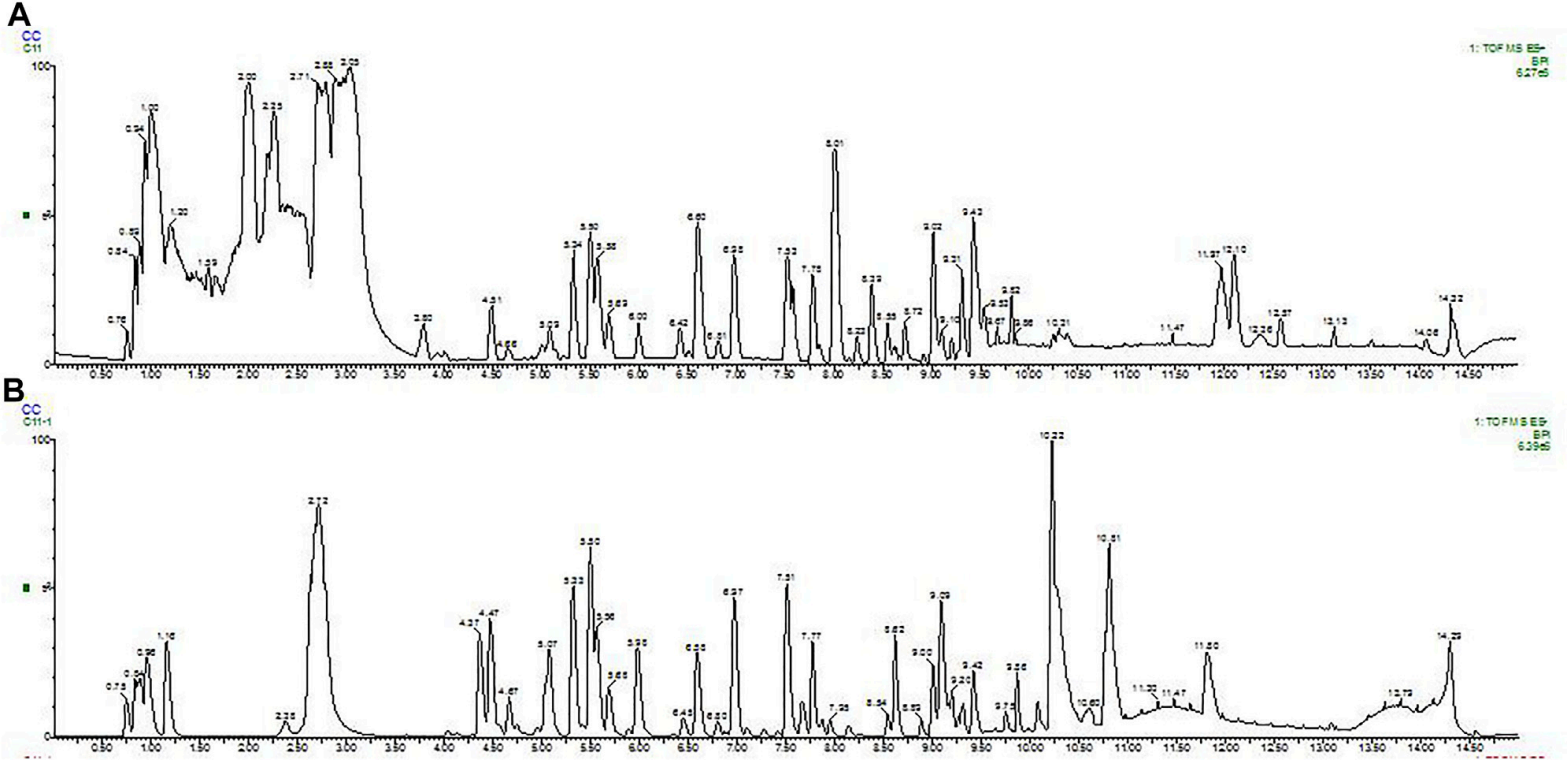
BPI (base peak intensity) diagram of fruit. A: Positive ion BPI (base peak intensity) diagram of fruit; B: Negative ion BPI (base peak intensity) diagram of fruit.

A total of 78 compounds were identified in the SDE, which belong to phenols, alkaloids, flavonoids, etc., the flavonoids include prenylated flavonols, prenylated isoflavonoids, isoflavone and chalcone, etc (Figure 1 and Table 2).

**TABLE 2:** The retention time and MS data of 78 compounds in fruit detected by UPLC–MS/MS.

No	Compounds	RT (min)	Molecular formula	Molecular weight	[M + H]	[M–H]	[M + Na]
1	Sophoramine	0.726	C_15_H_21_N_2_O	245.1654	√	—	—
2	Alopecurone A	0.780	C_39_H_37_O_9_	649.2438	—	√	—
3	Kushenol W	0.815	C_21_H_21_O_7_	385.1287	—	√	—
4	Cinnamic acid	0.841	C_9_H_7_O_2_	147.0446	—	√	—
5	Alopecurone B	0.866	C_39_H_37_O_9_	649.2438	—	√	—
6	Quercetin	0.892	C_15_H_11_O_7_	303.0505	√	—	—
7	Sophocarpine	0.936	C_15_H_23_N_2_O	247.1810	√	—	—
8	Kaushenol N	0.954	C_26_H_31_O_7_	455.2070	√	—	—
9	Daidzein	0.963	C_15_H_13_O_4_	257.0814	—	√	—
10	Sparteine	0.989	C_15_H_27_N_2_	235.2174	√	—	—
11	Aloperine	1.049	C_15_H_25_N_2_	233.2018	√	—	—
12	Secundiflorol A	1.155	C_21_H_23_O_8_	403.1393	√	—	—
13	Kushenol L	1.312	C_25_H_29_O_7_	441.1913	—	√	—
14	Leachianone G	1.460	C_20_H_21_O_6_	357.1338	√	—	—
15	Sophoraisoflavanone A	1.521	C_20_H_18_O_6_	353.1025	√	—	—
16	Kushenol B	1.547	C_30_H_37_O_6_	493.2590	√	—	—
17	Caffeic acid	1.573	C_9_H_9_O_4_	181.0501	√	—	—
18	Gallic acid	1.609	C_7_H_5_O_5_	169.0137	—	√	—
19	Luteoloside	1.686	C_21_H_21_O_11_	449.1084	—	√	—
20	Kaushenol H	2.018	C_26_H_31_O_7_	455.2070	√	—	—
21	Benzoic acid	2.237	C_7_H_5_O_2_	121.0290	—	√	—
22	Ferulic acid	2.534	C_10_H_9_O_4_	193.0501	—	√	—
23	N-Methylaytisine	3.773	C_12_H_16_N_2_O	205.1341	√	—	—
24	LupinifoLin	3.957	C_25_H_25_O_5_	405.1702	—	√	—
25	Secundiflorol D	4.035	C_21_H_23_O_6_	371.1495	√	—	—
26	Kushenol Q	4.183	C_25_H_29_O_7_	441.1913	—	√	—
27	Rutin	4.315	C_27_H_29_O_16_	609.1456	—	√	—
28	Daidzin	4.324	C_21_H_21_O_9_	417.1186	√	—	—
29	Matrine	4.375	C_15_H_25_N_2_O	249.1967	√	—	—
30	Sophoraisoflavanone D	4.402	C_30_H_37_O_6_	493.2590	√	—	—
31	Vicenin–2	4.428	C_27_H_29_O_15_	593.1509	—	√	—
32	Sophoricoside	4.515	C_21_H_19_O_10_	431.0978	—	√	—
33	Isokurarinone	4.550	C_26_H_29_O_6_	437.1964	—	√	—
34	Kurarinol	4.576	C_26_H_31_O_7_	455.2070	—	√	—
35	Saponarin	4.585	C_27_H_29_O_15_	593.1509	—	√	—
36	Isosakuranin	4.594	C_22_H_23_O_10_	447.1291	—	√	—
37	Sophoridine	4.595	C_15_H_25_N_2_O	249.1967	√	—	—
38	Kushenol X	4.638	C_25_H_29_O_7_	441.1913	—	√	—
39	Echinoisoflavanone	4.655	C_22_H_25_O_7_	401.1600	√	—	—
40	(^2^S) –2′–methoxykurarinone	4.733	C_27_H_33_O_6_	453.2277	√	—	—
41	Chlorogenic acid	4.917	C_16_H_17_O_9_	353.0873	—	√	—
42	Kushenol C	4.934	C_25_H_25_O_7_	437.1600	—	√	—
43	Triterpene	4.943	C_22_H_21_O_10_	445.1135	—	√	—
44	Kurarinone	4.943	C_26_H_29_O_6_	437.1964	—	√	—
45	Alopecurone D	4.952	C_40_H_39_O_9_	663.2594	—	√	—
46	Epicatechin	4.978	C_15_H_13_O_6_	289.0712	—	√	—
47	Erythrabrssin II	4.987	C_25_H_29_O_4_	393.2066	√	—	—
50	Kuraridinol	5.178	C_26_H_31_O_7_	455.2070	—	√	—
51	Diosmetin	5.589	C_16_H_13_O_6_	301.0712	√	—	—
52	Cytisine	5.702	C_11_H_14_N_2_ONa	213.1004	—	—	√
53	Isoquerctrin	5.807	C_21_H_19_O_12_	463.0877	—	√	—
54	Exiguaflavanone D	6.042	C_30_H_37_O_7_	509.2539	—	√	—
55	Sophpraflavanone B	6.504	C_20_H_19_O_5_	339.1232	—	√	—
56	Kushenol K	6.889	C_27_H_31_O_6_	483.2019	—	√	—
57	Kushenol U	7.099	C_25_H_29_O_5_	409.2015	—	√	—
58	Luteolin	7.508	C_15_H_9_O_6_	285.0399	—	√	—
59	Alopecurone J	8.198	C_39_H_37_O_9_	649.2438	—	√	—
60	Alopecurone K	8.609	C_39_H_37_O_9_	649.2438	—	√	—
61	Sophoraflavanone I	8.949	C_39_H_37_O_9_	649.2438	—	√	—
62	Sophoranone	8.992	C_30_H_35_O_5_	475.2484	—	√	—
63	Maackiain	9.323	C_16_H_11_O_5_	283.0606	—	√	—
64	Kushenol T	9.324	C_25_H_31_O_6_	427.2121	√	—	—
65	Norkurarinone	9.630	C_25_H_29_O_6_	425.1964	√	—	—
66	Leachianone B	9.672	C_26_H_29_O_6_	437.1964	—	√	—
67	Leachianone A	9.962	C_26_H_29_O_6_	437.1964	—	√	—
68	Sophoraflavanone H	10.179	C_34_H_31_O_9_	583.1968	√	—	—
69	Alopecurone G	10.240	C_26_H_29_O_5_	421.1025	—	√	—
70	Flavenochromane C	10.764	C_24_H_25_O_6_	421.1615	—	√	—
71	Secundiflorol E	10.765	C_22_H_25_O_7_	401.1600	√	—	—
72	Isosophoranone	10.807	C_26_H_29_O_6_	437.1964	—	√	—
73	8–Lavandulylkaempferol	10.948	C_24_H_25_O_6_	421.1615	—	√	—
74	Orientin	11.514	C_21_H_19_O_11_	447.0927	—	√	—
75	Sophpraflavanone G	12.073	C_25_H_27_O_6_	423.1808	—	√	—
76	Desmethylanhydroicaritin	12.222	C_20_H_17_O_6_	353.1025	—	√	—
77	Daucosterol	13.984	C_35_H_59_O_6_	575.4312	—	√	—
78	Kushenol S	14.289	C_20_H_19_O_5_	339.1232	—	√	—

The UPLC based quantitative analysis of polyphenols and alkaloids further indicated that the contents of oxysophocarpine and caffeic acid were the highest among the alkaloids and polyphenols, respectively (Table 3).

**TABLE 3:** Quantification of major polyphenols and alkaloids in SDE.

Compound	Content (mg/g·SDE)
*p*-Hydroxybenzoic acid	0.026 ± 0.07^ab^
Caffeic acid	1.911 ± 0.29^g^
Epicatechin	0.101 ± 0.65^cd^
Rutin	0.077 ± 0.10^bcd^
Ferulic acid	0.011 ± 0.03^a^
Quercetin	1.248 ± 0.43^f^
Cytisine	2.142 ± 0.01^abcd^
Oxysophoridine	0.070 ± 0.00^abc^
Matrine	0.127 ± 0.01^d^
Sophoridine	0.363 ± 0.01^e^
Sophocarpine	0.116 ± 0.00^d^
Oxymatrine	17.736 ± 0.23^g^
Oxysophocarpine	24.951 ± 0.52^h^

Different lowercase letters represent significant differences (*p* < 0.05). mg/g: Milligrams of a certain compound contained in each Gram of dry SDE.

### Mice Body Weight Change and Organ Index

During the D–gal–induction, the growth and development of mice (Table 4) and their health condition were documented. There were no difference in the weights among groups at the beginning of the experiment, while after 4 weeks of continuous feeding, the weight gain in each group increased in varying degrees, in which, D–gal group mice slowed the increase, accompanied with mental atrophy, slow movement, rough hair, reduced activity, and other general behavioral states; mice in the SL-, SM-, SH- and Vc group had significant weight gains, and in a dose-dependent manner among SDE groups.

**TABLE 4:** Changes in body weight during feeding.

Group	Weight (g)					
	0 weeks	1 week	2 weeks	3 weeks	4 weeks	Weight Gain
NC	31.79 ± 1.50	31.93 ± 1.06	33.24 ± 1.07	33.92 ± 1.35	34.29 ± 1.68	2.50 ± 0.72
D–gal	32.33 ± 1.26	33.27 ± 2.35	33.40 ± 2.32	34.42 ± 2.18	34.00 ± 1.95	1.67 ± 0.23
SL	32.97 ± 0.81	33.57 ± 1.16	34.22 ± 1.66	34.29 ± 1.58	34.90 ± 2.66	1.93 ± 0.99
SM	30.76 ± 1.13	31.40 ± 1.28	31.73 ± 1.77	32.33 ± 2.17*	32.86 ± 2.01	2.10 ± 0.26
SH	31.50 ± 0.79	32.02 ± 0.91	32.39 ± 0.94	33.14 ± 1.43	34.17 ± 1.31	2.67 ± 0.72*
Vc	31.40 ± 0.75	32.29 ± 1.48	32.88 ± 1.60	33.07 ± 1.43	34.31 ± 1.40	2.91 ± 0.86*

Compared with D–gal group, *: *p* < 0.05. NC, normal control group; D–gal, D–gal model group; SL, low–dose SDE, group; SM, medium–dose SDE, group; SL, high–dose SDE, group; Vc, Vc positive control group.

The organ index is calculated by dividing the weight of the organ by the weight of the mouse. The liver and brain indexes of mice in the D–gal group were dramatically lower than those in the NC group (*p* < 0.05), while the indexes were significantly higher in SM, SH and Vc groups than in D–gal group (*p* < 0.05, Table 5).

**TABLE 5:** Mice organ index.

Group	Liver index (%)	Brain index (%)
NC	4.001 ± 0.141	1.098 ± 0.038
D–gal	3.514 ± 0.465^#^	0.999 ± 0.073^#^
SL	3.773 ± 0.187	1.018 ± 0.055
SM	3.861 ± 0.161*	1.087 ± 0.096
SH	4.141 ± 0.157*	1.087 ± 0.082
Vc	4.007 ± 0.341*	1.089 ± 0.061

Compared with normal group, #: *p* < 0.05; Compared with D–gal group, *: *p* < 0.05. NC, normal control group; D–gal, D–gal model group; SL, low–dose SDE, group; SM, medium–dose SDE, group; SL, high–dose SDE, group; Vc, Vc positive control group.

### Histological Changes

**FIGURE 2:**
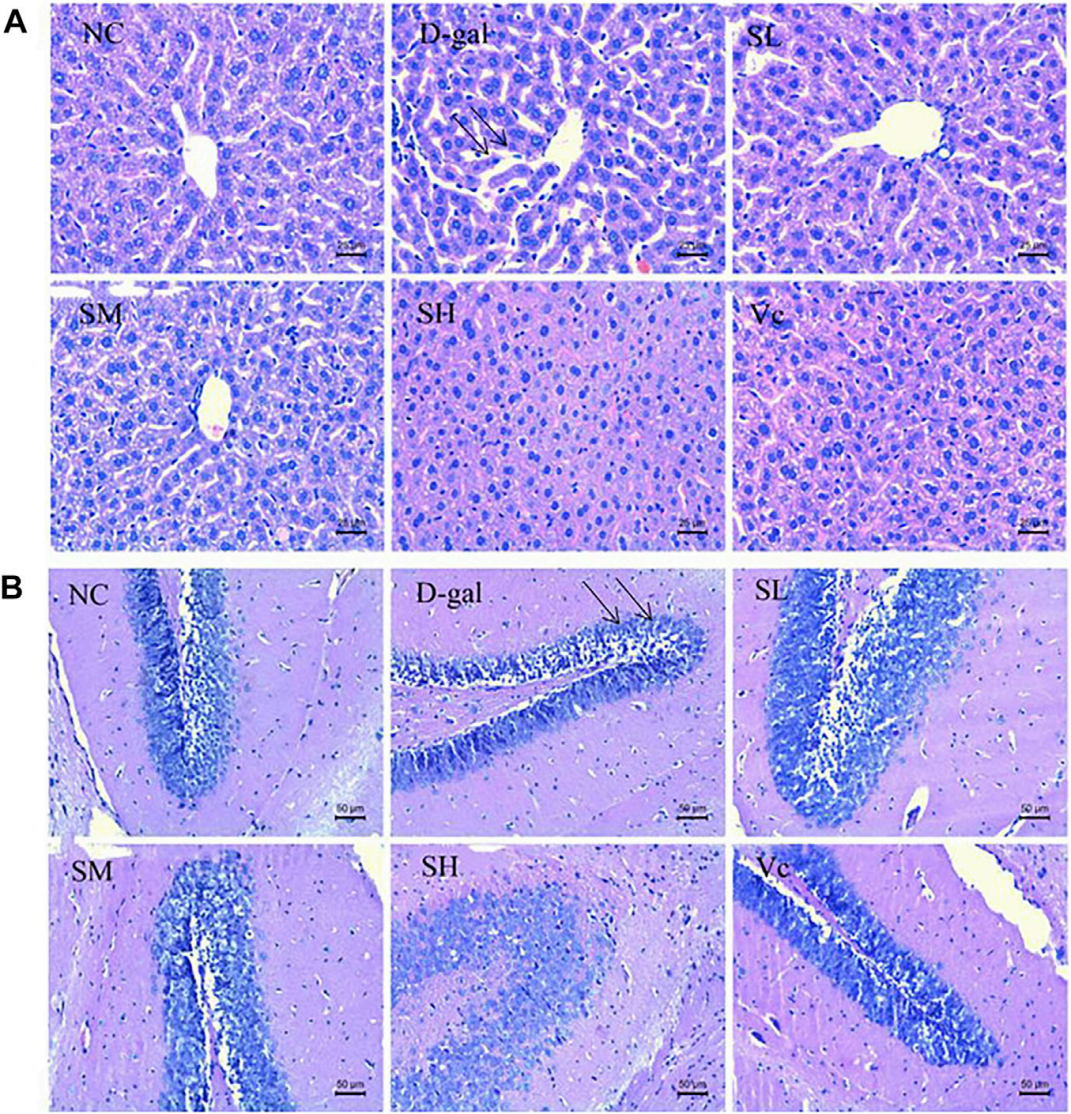
Histopathological changes of liver **(A)** and brain **(B)** in mice (×400). NC: normal control group; D–gal: D–gal model group; SL: low–dose SDE group; SM: medium–dose SDE group; SL: high–dose SDE group; Vc: Vc positive control group. The arrow indicates the injury site.

As shown in Figure 2A, the normal structure and obvious boundary of hepatocytes appeared without congestion in the liver of normal mice. The liver cells in D–gal group were disorganized; the intercellular space was enlarged (indicated by the arrow) and ballooning degeneration; the nuclear staining was deepened; some hepatocytes were missing; and there was obvious liver damage. After treatment, the SL and SM groups have basically improved, SH and Vc groups have no significant difference compared with the NC group, indicating that SDE had an improving effect on the D–gal–induced liver injury in aging mice.

Figure 2B shows the pathological changes of the hippocampus in the brain of mice. The neurons in the hippocampus of the normal group were closely arranged, with a large number, complete morphology and no necrosis. In the D–gal group, the neurons in the hippocampus were sparse (indicated by the arrow) and stained deeply, and necrotic cells appeared. The arrangement of cells in SL group and SM group was loose, with a small amount of necrosis and damage of hippocampal cells, the arrangement of hippocampal cells in SH group and Vc group was orderly and compact, with complete morphology. SDE could inhibit the apoptosis of hippocampal neurons and improve the D–gal–induced brain aging.

### iNOS Activity in Serum

**FIGURE 3:**
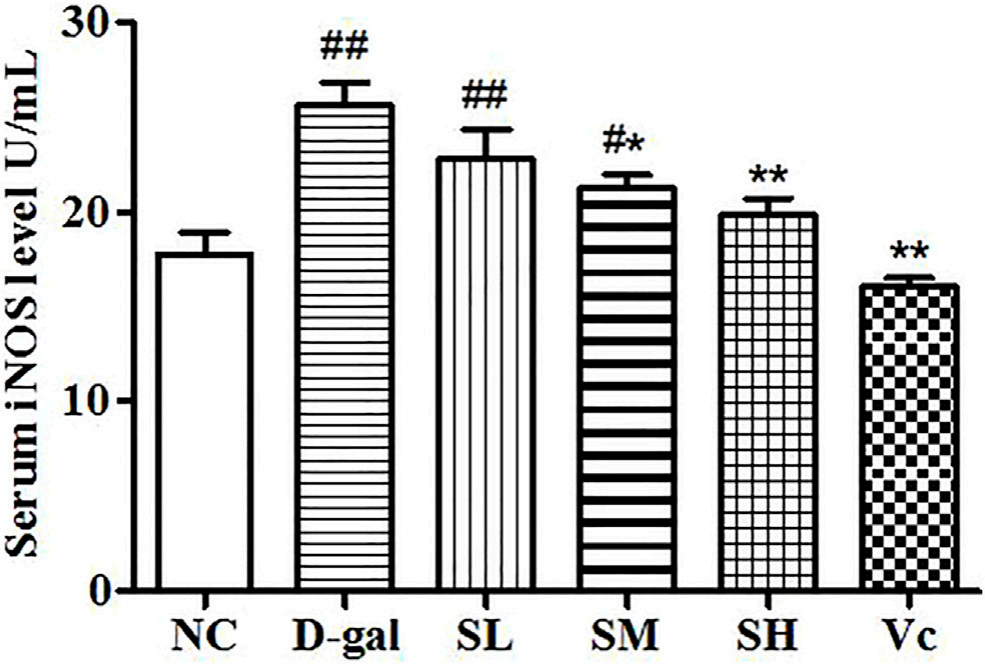
Serum iNOS level. Compared with normal group, #: *p* < 0.05, ##: *p* < 0.01; Compared with model group, *: *p* < 0.05, **: *p* < 0.01. NC: normal control group; D–gal: D–gal model group; SL: low–dose SDE group; SM: medium–dose SDE group; SL: high–dose SDE group; Vc: Vc positive control group.

Compared with the NC group, the level of iNOS in the D–gal group increased significantly (*p* < 0.01; Figure 3) and the activity of iNOS in the SL group decreased, but there was a significant difference between the NC group and SL group (*p* < 0.01). Compared to the D–gal group, the iNOS activity in the SH group and the Vc group decreased significantly (*p* < 0.01).

### AChE Activity in the Brain

**FIGURE 4:**
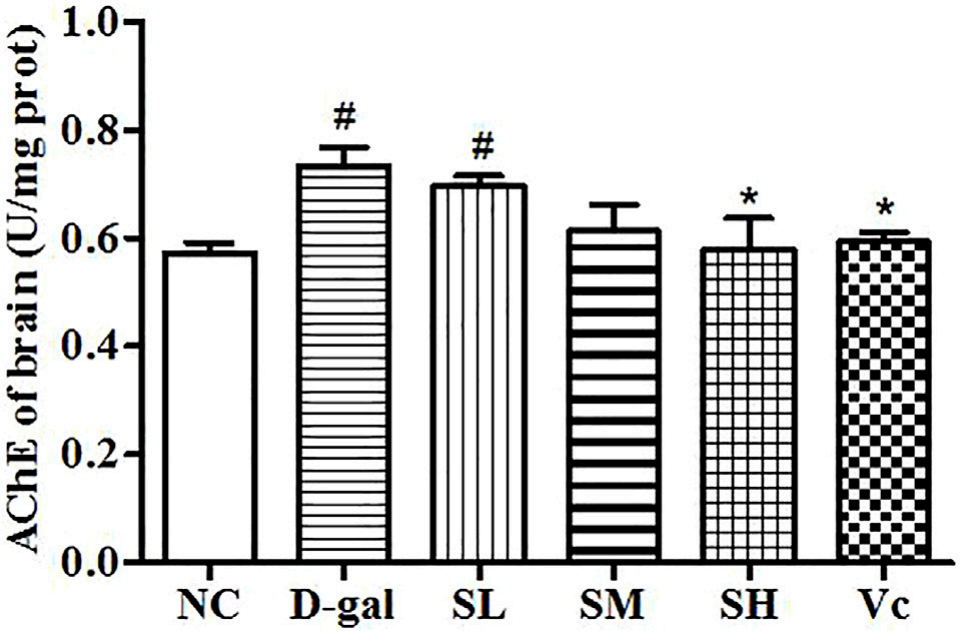
Activity of AChE in mice brain. Compared with normal group, #: *p* < 0.05; Compared with D–gal group, *: *p* < 0.05. NC: normal control group; D–gal: D–gal model group; SL: low–dose SDE group; SM: medium–dose SDE group; SL: high–dose SDE group; Vc: Vc positive control group.

AChE activity increased significantly in the D–gal group (*p* < 0.05; Figure 4), indicating the success of modeling. The AChE activity in the midbrain of the SH group and the Vc group decreased significantly and returned to the normal level (*p* < 0.05). However, AChE activities decreased in the SL and SM groups, despite no significant differences compared with the NC group. These results suggest that SDE may delay brain aging by improving the function of the cholinergic system.

### MDA in the Liver and Brain

**FIGURE 5:**
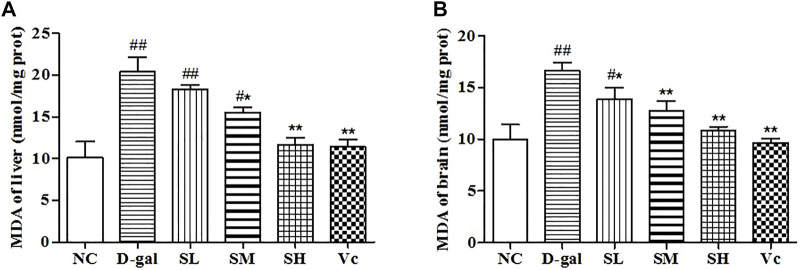
The MDA content of liver **(A)** and brain **(B)** in mice. Compared with normal group, #: *p* < 0.05, ##: *p* < 0.01; Compared with D–gal group, *: *p* < 0.05, **: *p* < 0.01. NC: normal control group; D–gal: D–gal model group; SL: low–dose SDE group; SM: medium–dose SDE group; SL: high–dose SDE group; Vc: Vc positive control group.

D–gal could induce the increase of MDA content in liver and brain (*p* < 0.01; Figure 5). The MDA content in the liver and brain decreased after the treatment with SDE, and there was a significant difference between the SH group, Vc group, and D–gal group (*p* < 0.01).

### GSH, SOD and T–AOC Level in the Liver and Brain

**FIGURE 6:**
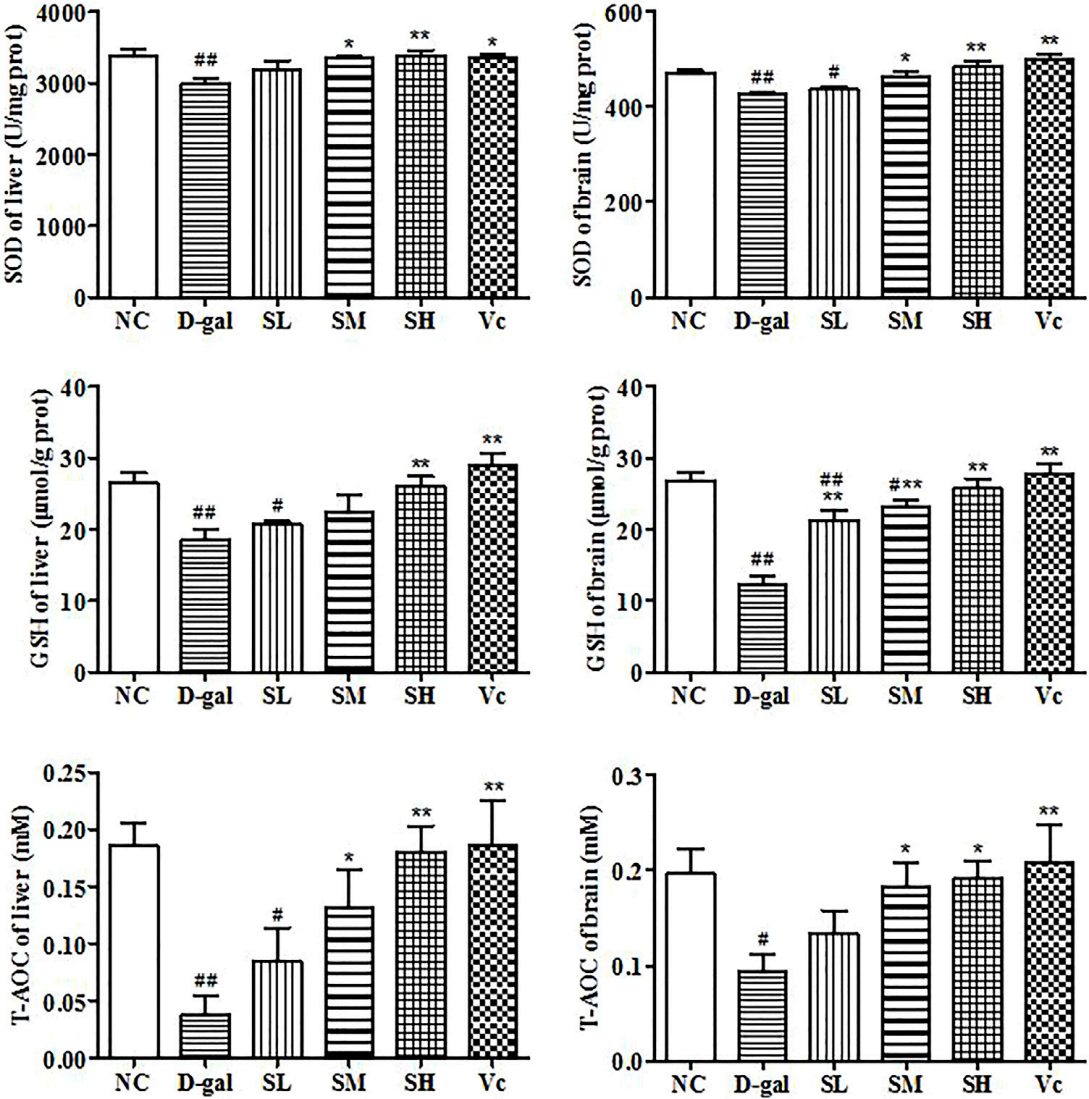
Antioxidant enzyme activity in liver and brain. Compared with normal group, #: *p* < 0.05, ##: *p* < 0.01; Compared with D–gal group, *: *p* < 0.05, **: *p* < 0.01. NC: normal control group; D–gal: D–gal model group; SL: low–dose SDE group; SM: medium–dose SDE group; SL: high–dose SDE group; Vc: Vc positive control group.

In this study, we investigated the activities of SOD, T–AOC and GSH (Figure 6). Long-term injection of D–gal in the liver and brain reduced significantly GSH level (*p* < 0.01), SOD and T–AOC activity (*p* < 0.05). However, when the mice were treated with SDE, antioxidant enzyme levels in mice gradually return to normal. These results suggested that SDE treatment could improve significantly the antioxidant stress of the liver and brain.

### SIRT1 and p53 Protein Expression in the Liver

**FIGURE 7:**
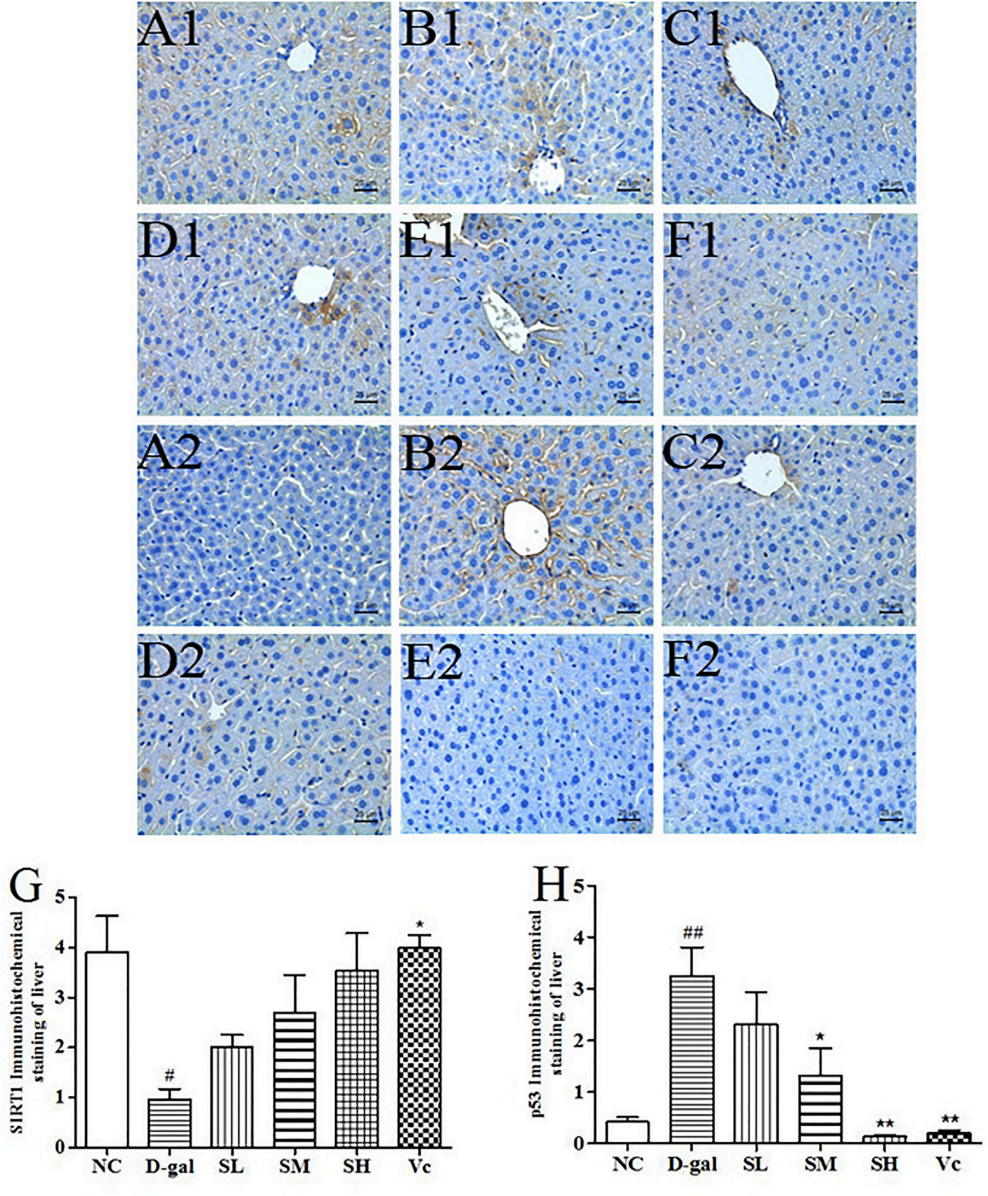
The SIRT1 and p53 protein expressions in the liver tissues of each group (×400). SIRT1 (A1–F1), A1: NC group, B1: D–gal group, C1: low–dose group, D1: medium–dose group, E1: high–dose group, F1: Vc group; p53 (A2–F2), A2: NC group, B2: D–gal group, C2: low–dose group, D2: medium–dose group, E2: high–dose group, F2: Vc group. G: The SIRT1 positive staining area in each group; H: The p53 positive staining area in each group. Compared with normal group, #: *p* < 0.05; ##: *p* < 0.01; Compared with D–gal group, *: *p* < 0.05, **: *p* < 0.01. NC: normal control group; D–gal: D–gal model group; SL: low–dose SDE group; SM: medium–dose SDE group; SL: high–dose SDE group; Vc: Vc positive control group.

The SIRT1 protein expression levels in the D–gal group were significantly reduced (*p* < 0.05), as compared with those of the mice in the NC group (Figure 7G). After treatment with SDE, the SIRT1 protein expression levels was increased in the mice of SL and SM and SH groups, and a dose-dependent manner. The p53 protein expression levels in the D–gal group were 676.19% higher than that of the NC group (Figure 7H). The SL, SM, SH and Vc groups can significantly reduce the expressions of p53 protein in the liver of aging mice. The anti-aging effect of SDE may be related to the activation of a SIRT1/p53 signal pathway.

### SIRT1 and p53 Protein Expression in the Brain

**FIGURE 8:**
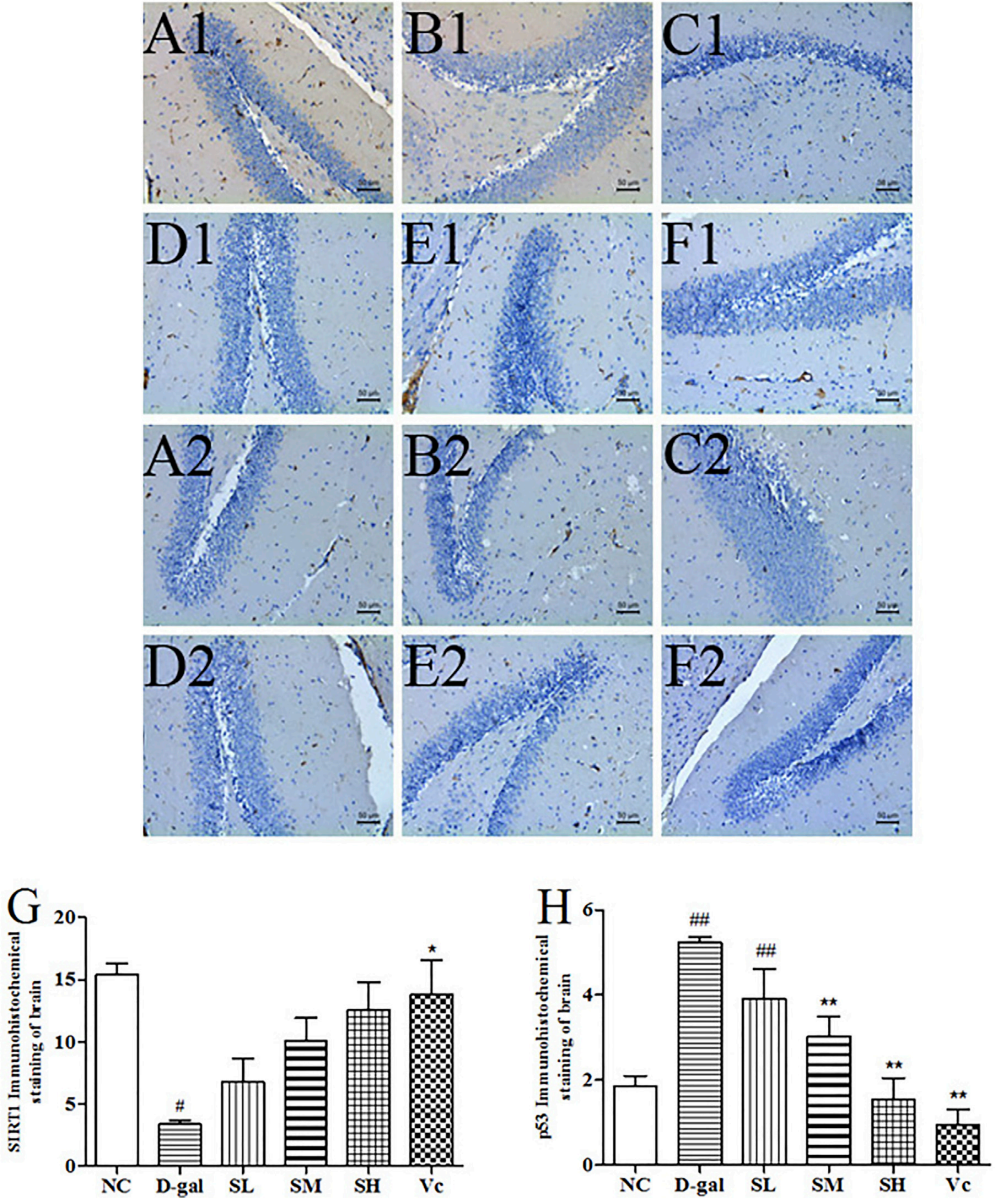
The SIRT1 and p53 proteins expression in the brain tissues of each group (×200). SIRT1 (A1–F1), A1: NC group, B1: D–gal group, C1: low–dose group, D1: medium–dose groups, E1: high–dose group, F1: Vc group; p53 (A2–F2), A2: NC group, B2: D–gal group, C2: low–dose group, D2: medium–dose group, E2: high–dose group, F2: Vc group. G: The SIRT1 positive staining area in each group; H: The p53 positive staining area in each group. Compared with normal group, #: *p* < 0.05, ##: *p* < 0.01; Compared with D–gal group, *: *p* < 0.05, **: *p* < 0.01. NC: normal control group; D–gal: D–gal model group; SL: low–dose SDE group; SM: medium–dose SDE group; SL: high–dose SDE group; Vc: Vc positive control group with normal group, #: *p* < 0.05, ##: *p* < 0.01; Compared with D–gal group, *: *p* < 0.05, **: *p* < 0.01. NC: normal control group; D–gal: D–gal model group; SL: low–dose SDE group; SM: medium–dose SDE group; SL: high–dose SDE group; Vc: Vc positive control group.

D–gal significantly decreased SIRT1 levels (Figure 8G, *p* < 0.05) and increased the expression of p53 protein in the brain (Figure 8H, *p* < 0.01). The SIRT1 protein expression significantly reduced (*p* < 0.05) in the D-gal group, while SDE treatment significantly increased SIRT1 in a dose-dependent manner. After Vc treatment, SIRT1 protein expression was higher than in the SH groups, and there was significant difference compared with the D–gal group (*p* < 0.05). The expression of p53 protein was attenuated by SDE in a dose-dependent manner.

### mRNA Expressions of TNF–α, NF–kB, IL–1β, IL–6 and Klotho in the Liver

**FIGURE 9:**
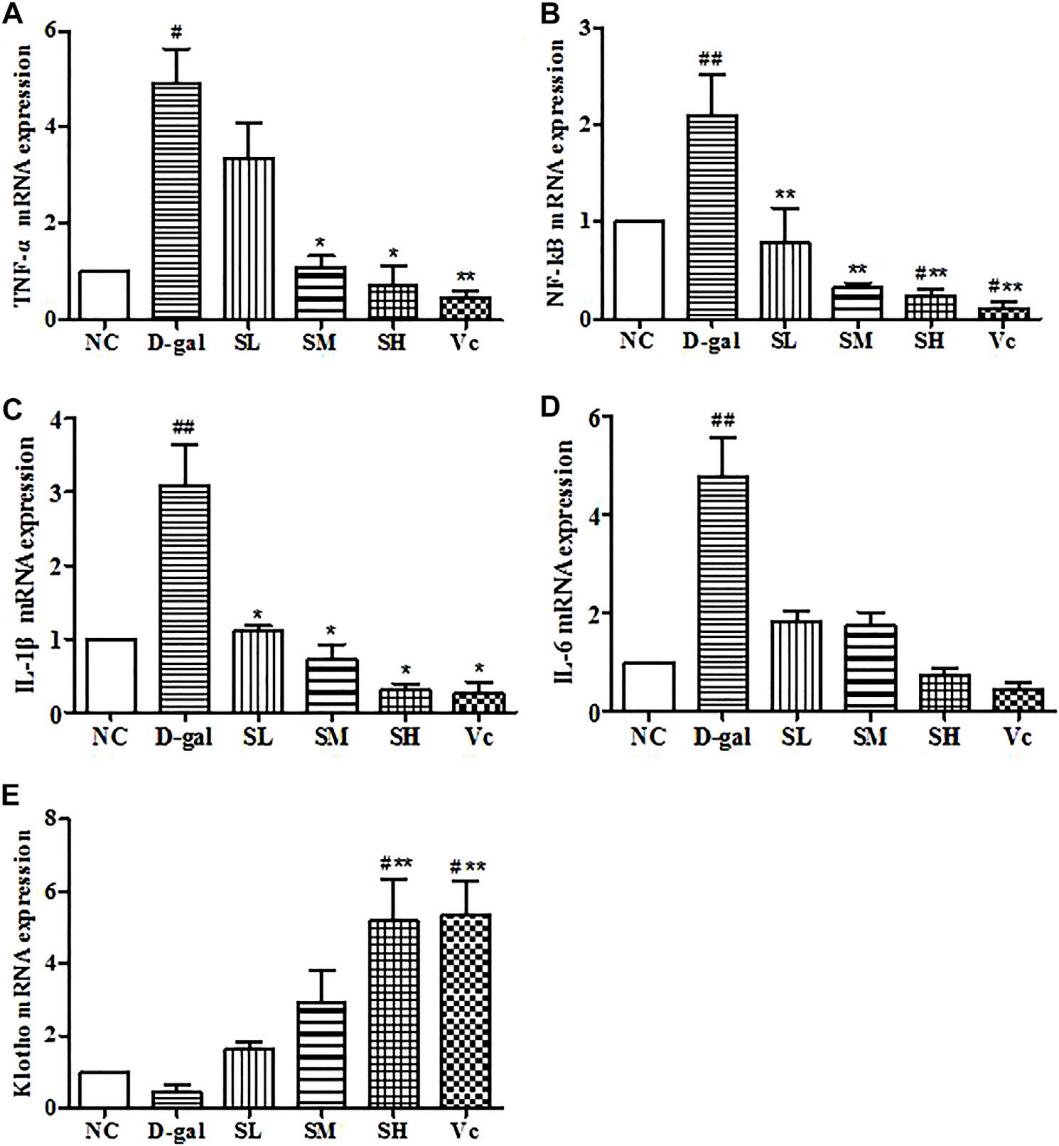
mRNA levels of TNF–α, NF–kB, IL–1β, IL–6 and Klotho in mice liver. Compared with normal group, #: *p* < 0.05, ##: *p* < 0.01; Compared with D–gal group, *: *p* < 0.05, **: *p* < 0.01. NC: normal control group; D–gal: D–gal model group; SL: low–dose SDE group; SM: medium–dose SDE group; SL: high–dose SDE group; Vc: Vc positive control group.

The mRNA expression of TNF–α, NF–kB, IL–1β and IL–6 significantly increased (*p* < 0.05) in the D-gal group, while SDE treatments remarkably decreased TNF–α, NF–kB, and IL–1β levels (Figures 9A–C). The expression of Klotho mRNA decreased in the D–gal group (Figure 9E). After SDE treatments, the expression of Klotho mRNA increased in the SL and the SM groups. There was a significant difference among SH, Vc, and NC groups (*p* < 0.05), and there was a very significant difference compared with the D–gal group (*p* < 0.01). These results indicate that SDE can reverse the high expression of inflammatory factors in the liver of aging mice.

### mRNA Expressions of TNF–α, NF–kB, IL–1β, IL–6 and Klotho in the Brain

**FIGURE 10:**
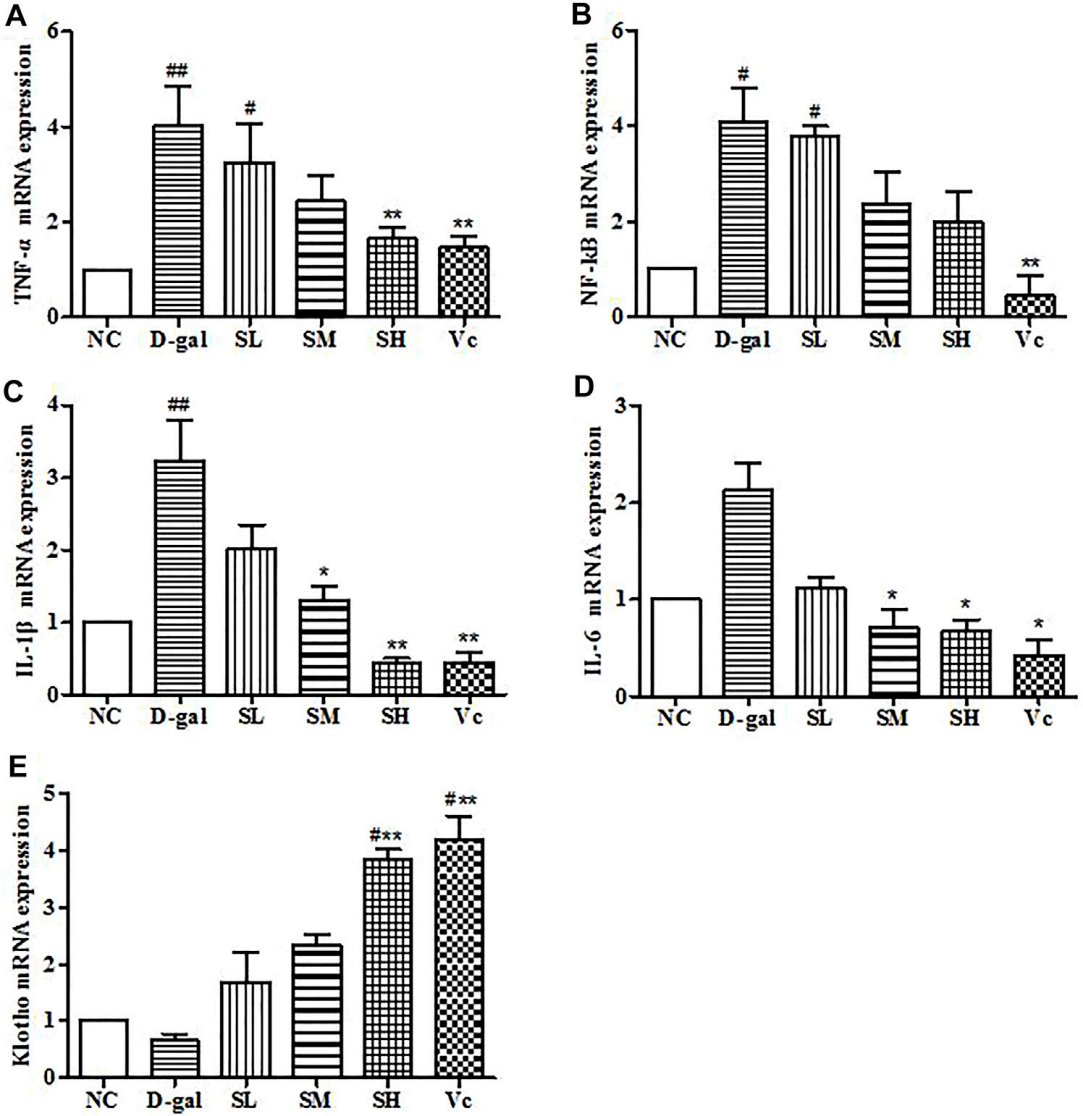
mRNA levels of TNF–α, NF–kB, IL–1β, IL–6 and Klotho in mice brain. Compared with normal group, #: *p* < 0.05, ##: *p* < 0.01; Compared with D–gal group, *: *p* < 0.05, **: *p* < 0.01. NC: normal control group; D–gal: D–gal model group; SL: low–dose SDE group; SM: medium–dose SDE group; SL: high–dose SDE group; Vc: Vc positive control group.

In the D–gal group, the mRNA expressions of TNF–α, NF–kB, and IL–1β increased significantly (*p* < 0.05). The mRNA expression of IL–6 increased, but the difference was not significant, which indicating that there was neuroinflammation in the brain. After treatment with SDE, the mRNA of TNF–α, NF–kB, IL–1β, and IL–6 were reduced significantly (*p* < 0.05; Figures 10A–D). Klotho mRNA expression in the D–gal group decreased, but the difference was not significant compared with the normal group (Figure 10E). The mRNA expression of Klotho increased in the SL group and the Vc group, which was different from the NC group (*p* < 0.05), and significantly different from the D–gal group (*p* < 0.01). The above shows that SDE can reduce neuroinflammation in the brain of aged mice.

## Discussion

*S. davidi* is a medicinal plant used in Chinese ethnic minorities. In our study, 78 compounds were initially identified in SDE, including flavonoids, polyphenols and alkaloids, in which, desmethlyanhydroicaritin, 8-lavandulylkeampferol and kushenol C had been confirmed with clear antioxidant activity *in vivo* (Boozari et al., 2018).

The iNOS can be induced or stimulated by various cytokines such as TNF-α, IL–1β, and IL-6. Under inflammatory conditions and oxidative stress, iNOS protein expression and iNOS gene transcription can be upregulated and remain active to produce enormous NO (Abdul et al., 2021). NO has been associated with the pathogenesis and progression of several diseases, such as liver diseases, insulin resistance, obesity and diseases of the cardiovascular system (Anavi and Tirosh, 2020). Therefore, inhibiting the production of iNOS and NO can inhibit the production of pro-inflammatory mediator factors. The iNOS activity in the D–gal group increased, and the mRNA expression of TNF–α and IL–1β in liver and brain tissues increased significantly, indicating that the mRNA expression of TNF–α and IL–1β increased. The body produces an inflammatory response, which raises the iNOS level, causing damage to the body. Excessive iNOS levels in the body can also trigger an inflammatory response. Our results suggested that SDE can reduce iNOS levels in mice, and consistent with previous reports (Zeng et al., 2018). AChE is a hydrolytic enzyme present mainly in the nervous system, which catalyzes the hydrolysis of the neurotransmitter acetylcholine (ACh). The cognitive ability of human and animal brain learning and memory are closely related to the function of the cholinergic system. AChE is a specific protease reflecting the function of the cholinergic system. It can degrade ACh in synaptic space and reduce its content. Therefore, increasing the level of ACh in the brain and decreasing the activity of AChE, thus enhancing the function of the cholinergic system in the brain may be one of the mechanisms of action against anti-aging and improving the brain’s ability for learning and memory. In the D–gal group, the AChE activity in the brain tissue significantly increased, which is consistent with the results of the study by Zhou (Zhou et al., 2013) and his colleagues. After treatment with SDE and Vc, AChE activity in the SH and the Vc group were significantly lower. Decreased AChE activity in brain, suggests that SDE may slow brain aging by improving the cholinergic system.

MDA is a product of lipid peroxidation caused by free radicals in the body, which is often used as an index to evaluate aging (Kong et al., 2018); T–AOC can reflect the total antioxidant capacity of the human body (Zhao et al., 2018). SOD is an important antioxidant enzyme *in vivo*, which can effectively eliminate superoxide anion free radicals, reduce the production of MDA and free radical metabolites, and protect cells from damage (Kong et al., 2018). GSH is the most important non–enzymatic antioxidant in the body, and the amount of GSH is also an important measure of the antioxidant capacity. After the D–gal group was treated with SDE, MDA, GSH content, T–AOC level and SOD activity in the liver and brain were significantly different from the normal group. The content of MDA in liver and brain was downregulated and the content of GSH, the level of T–AOC, and the activity of SOD were upregulated. The results of this study are consistent with a recent publication (Zhao et al., 2018), indicating that SDE has the same anti-aging effect as compound walnut oil capsule.

SIRT1 is a nucleohistone deacetylase dependent on nicotinamide adenine dinucleotide (NAD+), which is involved in the regulation of many physiological processes such as aging, stress cells, DNA repair, and metabolism, etc. Under normal circumstances, SIRT1 protein is highly expressed *in vivo*. p53 is a tumor suppressor that can be activated by many stressors and induces apoptosis, cell cycle arrest, or aging, and also plays a major role in the aging process (Nicole and Ulrich, 2008; Tian et al., 2019). Studies have shown that the activation of SIRT1 has a significant inhibitory effect on the senescence regulator p53, thereby reducing apoptosis and delaying senescence (Li et al., 2018). In our study, we used the immunohistochemical method analyze the expression of SIRT1 and p53 proteins induced by D–gal in the liver and brain of aging mice. The experimental results showed that SDE could increase significantly the expression of SIRT1 protein in the liver and brain of aging mice and inhibit effectively the overexpression of p53 caused by D–gal. The results are similar to those of Tian and his colleagues (Tian et al., 2019). The above results indicated that SDE has good anti-aging effects, and its mechanism may be associated with the activation of a SIRT1/p53 signaling pathway.

Kim et al. found that the expressions of TNF–α, NF–kB, IL–1β and IL–6 are upregulated in the liver and brain tissue of aging mice, indicating the body had an inflammatory reaction (Kim et al., 2020). A previous study (Ruan et al., 2013) demonstrated for the first time that D–gal induces liver cell senescence accompanied by upregulation of pro-inflammatory cytokines. SDE can downregulate the high expression of four kinds of proinflammatory factors, alleviate the inflammatory reaction in liver and brain tissues, and thus play an anti–aging role. Klotho is a type of anti-aging gene. Deficiency of Klotho leads to shortened life span, atherosclerosis, osteoporosis, cognitive and memory impairment, and aging characteristics. In contrast, overexpression of Klotho can prolong life (Wang and Sun, 2009). In addition, studies have shown that Klotho is an important factor in regulating oxidative stress, apoptosis, and cell proliferation. The results of our study show that SDE can increase significantly the expression of the Klotho gene in the liver and brain and protect them from oxidative stress.

## Conclusions

The extract of *S. davidi* fruit has anti-aging effect by reducing the activity of iNOS in serum and AChE in the brain, increasing the activity of antioxidant enzymes in liver and brain tissue, weakening the inflammatory response, upregulating the expression of SIRT1 protein, and inhibiting the over expression of p53 protein. The activation of a SIRT/p53 signal pathway was observed. As a promising medicinal and functional plant, further exploration on SDE chemical compositions and their pharmacological basis are expected.

## Data Availability

The datasets presented in this study can be found in online repositories. The names of the repository/repositories and accession number(s) can be found in the article/Supplementary Material.
